# Placental Pathology and Blood Pressure's Level in Women with Hypertensive Disorders in Pregnancy

**DOI:** 10.1155/2012/684083

**Published:** 2012-05-07

**Authors:** Vassiliki Krielessi, Nikos Papantoniou, Ioannis Papageorgiou, Ioannis Chatzipapas, Efstathios Manios, Nikos Zakopoulos, Aris Antsaklis

**Affiliations:** First Department of Obstetrics and Gynecology, University of Athens, “Alexandra” Hospital, Lourou and Vasilissis Sofias, 11528 Athens, Greece

## Abstract

*Objective*. The aim of this study was to investigate the extent of placental lesions associated with blood pressure (BP) levels in pregnancies complicated by hypertension. *Methods*. 55 singleton pregnancies complicated by mild hypertension were recruited and compared to 55 pregnancies complicated by severe hypertension. The histological assessment was carried out with regard to the following aspects: vessels number/field of vision, infarction, villous fibrinoid necrosis, villous hypermaturity, avascular villi, calcifications, lymphohistiocytic villitis, and thickened vessels. Statistical analysis was performed by SPSS. *Results*. All placental lesions were observed more often in the severe hypertension group. Vessels number was significantly decreased, and infarction and villous fibrinoid necrosis were significantly increased in the placentas of the severe hypertension group compared to the mild hypertension group (*P* < 0.001). *Conclusion*. This study supports that the extent of placental lesions in hypertensive pregnancies is correlated with hypertension level and so highlights blood pressure level as a mirror of placental function.

## 1. Introduction

The term gestational hypertension is used now to describe any form of new-onset pregnancy-related hypertension. There are five types of hypertensive disorders: (1) Gestational hypertension. (2) Preeclampsia. (3) Eclampsia. (4) Preeclampsia superimposed on chronic hypertension. (5) Chronic hypertension [1].

Hypertensive disorders complicating pregnancy are common and form one of the deadly triad, along with hemmorage and infection, that contribute greatly to maternal and fetal morbidity and mortality. Many studies estimate that hypertension is responsible for 2.6% to 7.6% of maternal deaths [2–5]. Pregnancies complicated with preeclampsia have a high incidence of preterm delivery [6], fetal growth restriction, low birth weight, placental abruption, caesarian delivery, liver insufficiency, subcapsular liver hematoma, cerebral edema, renal failure, thrombocytopenia, and intravascular coagulation.

The complications of hypertensive disorders in pregnancy have been attributed to abnormalities in the placenta [7, 8]. Therefore the placenta of the hypertensive woman has gained much interest, and several pathological changes have been described [9]. Placental villous angiogenesis may soon be viewed as central to normal perinatal development and survival [10].

The aim of this study was to test this hypothesis by investigating various macroscopic and microscopic morphological features of the placenta in pregnancies complicated by severe hypertension compared to pregnancies complicated by mild hypertension.

## 2. Materials and Methods

### 2.1. Study Design and Participants

In this prospective study the population at recruitment consisted of 323 pregnant women with hypertensive disorders as admission possible diagnosis. Inclusion criteria were singleton pregnancy, gestational hypertension, preeclampsia, eclampsia, preeclampsia superimposed on chronic hypertension, and chronic hypertension. These women were hospitalized in the high-risk pregnancy unit of “Alexandra” Maternity University Hospital, First department of Obstetrics and Gynaecology, in Athens. The protocol was approved by the hospital ethics committee, and informed consent was obtained from all pregnant women.

### 2.2. Clinic Blood Pressure Measurements

Clinic BP measurements were obtained by a mercury sphygmomanometer in all subjects three consecutive times. The first measurement was taken at the beginning of the visit, 5 minutes after sitting down; the second was taken 2 minutes later, and the third immediately before application of the ambulatory BP-monitoring equipment. The mean of the three systolic values and the mean of the three diastolic values of the same patient were the clinic systolic blood pressure (SBP) and the clinic diastolic blood pressure (DBP), respectively. Study participants remained seated with the arm comfortably placed at heart level, whereas measurements were obtained for each one by the same doctor, who was blinded to the study hypothesis.

Definitions of mild and severe hypertension are the following:

- Mild hypertension was defined as BP elevation of ≥140/90 mmHg on 2 measurements 4 hours apart during 24 h by sphygmomanometer [11–13].
- Severe hypertension was defined as BP elevation greater than 160/110 mmHg on 2 successive measurements 4 hours apart or one diastolic blood pressure of ≥110 mmHg during 24 h.

### 2.3. ABPM Measurements and Assessment

All subjects underwent ABPM for 24 hours with a Spacelabs 90207 ambulatory blood pressure monitor. The cuff was fixed to the nondominant arm with tape. Automatic blood pressure readings were taken at 15-minute intervals, and at least three valid BP measurements per hour over 24 hours were needed for the purpose of the study. Study participants were hospitalized during ABPM, but they were instructed to have usual activities (walking, sleeping, eating,…). The accuracy of ABPM devices was checked monthly by means of 10 automatic and 10 oscillatory BP readings taken simultaneously from the same arm via Y-tube. In all instances the values did not differ by >5 mmHg. The assessment of ABPM was performed at the hypertensive center of the Department of Clinical Therapeutics (Alexandra Hospital, Athens University, Greece).

### 2.4. Other Parameters Registration

In the women consisting the study group, age, gestational age, parity, weight gain, birthweight, gestational age at delivery, placenta weight, neonatal sex, and proteinuria were recorded. Proteinuria was defined as excretion of ≥300 mgr or 2 gr of protein in a 24-hour urine collection, apart with no evidence of urinary tract infection. Gestational age was assessed by the last menstrual period or by a first trimester ultrasound scan if there was a discrepancy of more than a week.

### 2.5. Pathologic Examination of Placenta Bed

A pathologist, blinded to all clinical data except of gestational age (in order to assess the villous maturation), reviewed all histological samples. The placentas were rinsed in water and left to drain of blood for at least 2 hours. In all cases the placenta was sliced into 2 cm thick coronal sections. The samples were controlled macroscopically for umbilical vessels abnormalities and calcifications. Representative specimens were taken from the cord insertion, full thickness of macroscopically normal placenta, peripheral part of placenta, placental membranes, and from abnormal areas. The placentas were weighed without umbilical cord. All samples were fixed in 10% formal-saline solution. After 24 hours embedded in paraffin, histological sections were taken by microtome (4 *μ*m). All samples were stained with hematoxylin and eosin. The biopsy specimens were examined by routine light microscopy (original magnification x40). We calculated the ratio (Fetal/Placental weight ratio) between placental weight and infant birth weight. The histological assessment was carried out with regard to the following aspects: villous hypermaturity, ischemia, lymphohistiocytic villitis, presence of avascular villi, massive perivillous fibrin deposition, villous fibrinoid necrosis, number of vessels per field of vision, vessel wall thickening and calcifications.

Definitions of main placental lesions are the following:

- Ischemia was defined as moderate when increased maturation and Tenney-Parker changes were present and severe when micro- or macroscopical infarcts were observed.
- Vessels' number/field of vision, as the number of vessels was calculated by a pathologist.
- Infarction, assessed at gross and microscopical examination.
- Villous fibrinoid necrosis, a condition where villous stroma is replaced with fibrinoid.
- Villous hypermaturity was defined when there was a numerous small terminal villi before the 40th week and numerous small cross-sections of highly capillarized terminal villi.
- Calcifications, assessed at gross examination.
- Lymphohistiocytic villitis was diagnosed by the presence of numerous lymphocytes and macrophages in the villous stroma.
- Avascular villi were diagnosed when a group of at least 5 fibrotic avascular villi without inflammation and without mineralization were seen.
- Thickened vessels were diagnosed by the presence of vascular stenosis and athirosis.

### 2.6. Statistical Analysis

Subjects were anonymised at recruitment, and data were entered on a database distribution table concerning categorical variables. Mean value, standard deviation, standard error, and 95% confidence interval described the continuous variables.

Correlation assessment between blood pressure groups and categorical variables of population was based on independence test *X* ^2^ (with continuity correction as necessary) or Fisher's test. Linear Pearson's *r* correlation coefficient was used for the evaluation between continuous variables of sample in two blood pressure groups. One-way variance analysis (ANOVA) combined with unequal variations correction or *t*-test was used for continuous variables distribution evaluation between blood pressure groups.

All tests were 2 sided, and level of statistical significance was set at 5%.

## 3. Results

Of  323 women, in whom blood pressure was found abnormal (SBP ≥ 140 mmHg or/and DBP ≥ 90 mmHg), by routine office (sphygmomanometer) measurements, 42 did not record abnormal hypertensive values by ABPM. Then, 42 were excluded because they are suspected for episodic or white coat hypertension, or autonomic dysfunction. Investigation was therefore limited to 281 women. Of 281 women, 186 (66.2%) recorded mild hypertension, and 95 (33.8%) recorded severe hypertension.

In the severe hypertension group A SBPmean (by ABPM) was 147.47 ± 7.40 (132.42–163 mmHg) and DBPmean 93.90 ± 6.53 (84.13–109.75 mmHg) whereas in the mild hypertension group B SBPmean was 132.45 ± 5.75 (126.88–139.38 mmHg) and DBPmean 83.08 ± 4.18 (80.50–89 mmHg) during 24 h.

Histological sections were taken from 55 placentas of the mild hypertension group B and from 55 placentas of the severe hypertension group A. Furthermore, statistical analysis took place on two groups.

The main characteristics of the population are presented in Table 1 (maternal, fetal, and placental data). No differences existed between the groups regarding maternal age. Maternal weight gain was oddly lower in group A (*P* = 0.025), probably because of nutrition management. Nulliparous women were significantly more in pregnancies complicated by severe hypertension than mild hypertension (group A = 76.36% versus group B = 60%, *P* = 0.013).

Gestational age at recruitment differed significantly between two groups (group A = 30.65 ± 4.40 weeks; group B = 32.38 ± 5.23 weeks; *P* = 0.006), possibly because of earlier onset of severe hypertension. Proteinuria greater than 300 mgr/24 h was presented in significantly more women of group A than group B (80% versus 65.45%, *P* < 0.001). Proteinuria greater than 2 gr was observed in significantly more women of group A than group B (38.18% versus 12.72%, *P* < 0.001). Preterm deliveries were significantly more in pregnancies complicated by severe hypertension (group A = 67.27%, group B = 38.18%, *P* < 0.001). The male/female ratio was significantly higher in group A (group A = 1.41, group B = 1.02, *P* < 0.001).

Fetal weights resulted significantly lower in the severe hypertension group A (*P* < 0.001). However, since gestational age at delivery was higher in mild hypertension pregnancies (35.66 ± 2.88 weeks) than severe hypertension pregnancies (34.04 ± 3.12), fetal weight remained significantly lower in severe hypertension group A after correcting for gestational age (1875.36 ± 675.19 versus 2057.79 ± 631.36, *P* < 0.01). Significantly lower placental weights (*P* < 0.001) were found in pregnancies complicated by severe hypertension and remained significantly lower after correction for gestational age (group A = 354.62 ± 122.33 gr, group B = 427 ± 137. 67 gr, *P* < 0.01). Fetal/placental weight ratios were significantly lower in group A.

The results of the histomorphological examination of placentas of women with severe hypertension (group A) compared with mild hypertension (group B) are shown in Table 2.

There were no cases of umbilical vessel abnormalities (numerical or morphological) and pathological findings from teguments between both groups. The number of vessels/field of vision was significantly decreased in group A (group A = 35.63 ± 8.47; group B = 43.6 ± 7.91; *P* < 0.001). The presence of infarction and villous fibrinoid necrosis was apparent, mainly in the severe hypertension group (*P* < 0.001). Villous hypermaturity was significantly more often in group A than group B (40% versus 25.45%, *P* = 0.051). Although avascular villi, calcifications, thickened vessels and lymphohistiocytic villitis were noted more often in the severe hypertension group than mild hypertension group, the results were not significantly different.

## 4. Discussion

Obstetrical and fetal or neonatal complications are common in pregnancies complicated with hypertensive disorders. It is believed that in these pregnancies impaired placental function, in terms of abnormal placental weight or histology, may account for this phenomenon. We assessed the relative placental weight and defined several histological abnormalities in placentas of mild and severe hypertension groups. Histological abnormalities such as the presence of villous fibrinoid necrosis, villous hypermaturity, and placental infarction were observed significantly more often in the hypertensive placentas, which is in agreement with other studies [9, 14, 15]. Placental dysfunction may affect oxygen exchange and lead to a state of oxidative stress and chronic-fetal hypoxemia [7]. Placental infarctions are the most common placental lesions, and their presence is a continuum from normal changes to extensive and pathological involvement. If they are numerous, thick, centrally located, and randomly distributed, placental insufficiency may develop [16]. Avascular villi, villitis are more frequent in preeclampsia [17]. Necrosis of villous tissue develops from ischemia. Histopathological features include fibrinoid degeneration of the trophoblast, calcification, and ischemic infractions.

The goal of this study was to correlate the extent of placental lesions with the level of hypertensive disorders. The conclusion was that significantly extensive placental lesions were associated with higher level of hypertension.

In the current study we found that the number of vessels/field of vision (magnification x40, standard micrometer) was significantly less in pregnancies complicated with severe hypertension than mild hypertension. This is consistent with other studies [12, 18, 19] although they have been referred in IUGR cases. Vascularization of the placental villi starts at day 21 after conception. From 26 weeks of gestation until term the villous vascular growth undergoes a change from branching to nonbranching angiogenesis. These specialized structures are the main site of diffusional gas and nutrition exchange between the maternal and fetal circulations. Consequently, fetoplacental blood flow is severely impaired and transplacental gas and nutrition exchange is poor, placing the fetus at risk of hypoxia, acidosis, and low birth weight.

In general, placental and fetal size and weight roughly correlate in a linear fashion. There is also evidence that fetal growth dependents on placental weight, which is less with small-for-gestational age infants [20]. When the rate of uteroplacental flow is chronically reduced, there is a tight direct linear correlation between the rate of mean uteroplacental blood flow and placental weight. The placenta has the ability to control the growth of the fetus. The mean birthweight and gestational age at delivery are lower, and preterm deliveries are higher in hypertension [21]. Birthweight is influenced from proteinuria. The presence of increased proteinuria predicted an adverse pregnancy outcome [22]. In this study gestational age at delivery, birthweight and placental weight after correcting for gestational age are less in the severe hypertension group than in the mild hypertension group, and so it confirms the hypothesis that the level of hypertension is correlated with the level of birthweight, placental weight, and gestational age at delivery. Our findings about birthweight, gestational age at delivery are consistent with other studies [4, 11].

ABPM is a useful diagnostic and therapeutic tool in hypertensive patients [23]. Automated BP measurement is more likely to identify patients conventionally characterized as hypertensive who have been misdiagnosed. The assessment of hypertensive pregnancies using ABPM appears to improve the ability to identify correctly patients who are at risk for poor obstetric outcome [24]. In the current study, 42 pregnant women, with abnormal office blood pressure measurements, did not record any blood pressure elevation greater than 140/90 mmHg by ABPM. These women are suspected for episodic hypertension, autonomic dysfunction [25] or white-coat phenomenon [26]. Although there are studies about women dissatisfaction, compliance problems, and sleep disturbance from ABPM devices [27, 28], there was not dissatisfaction nor compliance problems from the pregnant women after explaining to them in detail the benefits of ABPM.

## 5. Conclusion

Our findings support the hypothesis that impaired placental function accounts for hypertensive disorders in pregnancy. Moreover, we conclude that placental lesions in hypertensive pregnancies, such as infarctions, villous fibrinoid necrosis, and villous hypermaturity, are significantly correlated with hypertension severity. Furthermore, the placenta vascularization and angiogenesis are significantly poorer when the hypertension level is higher.

Neonatal and placenta weight are significantly lower in pregnancies complicated with severe hypertension compared with mild hypertension. Proteinuria and preterm deliveries are significantly more often in severe than mild hypertensive pregnancies.

## Figures and Tables

**Table 1:** Maternal, fetal and placental data.

	Group A (Severe hypertension)	Group B (Mild hypertension)
Maternal age (years)	32.01 ± 4.54	32.42 ± 5.46
Gestational age at recruitment (weeks)	30.65 ± 4.40**	32.85 ± 5.23
Weight gained (kgr)	11.88 ± 5.58*****	13.47 ± 6.61
Nulliparous	76.36%****	60**%**
Proteinuria > 300 mgr/24 h	80%*	65.45**%**
Proteinuria > 2 gr/24 h	38.18%*	12.72%
Gestational age at delivery (weeks)	34.04 ± 3.12*	35.66 ± 2.88
Fetal birth weight (gr)	1875.36 ± 675.19***	2057.79 ± 631.36
Placental weight (gr)	354.62 ± 122.33***	427 ± 137.67
Fetal/Placental weight ratios	5.53 ± 0.99******	5.98 ± 0.94
Preterm deliveries	67.27%*	38.18%
Male/female ratio	1.41	1.02

Mean ± s.d. *P < 0.001, **P = 0.006, ***P < 0.01, ****P = 0.013, *****P = 0.053, ******P = 0.025.

**Table 2:** Findings of histomorphological examination of placentas in severe hypertension group A versus mild hypertension group B.

	Group A Placentas (Severe hypertension)	Group B Placentas (Mild hypertension)
Vessels number/field of vision (mean ± s.d)	35.63 ± 8.47*	43.6 ± 7.91
Infarction	72.72%*	40%
Villous fibrinoid necrosis	72.72%*	32.72%
Villous hypermaturity	40%**	25.45%
Avascular villi	10.9%	7.27%
Calcifications	32.72%	25.45%
Lymphohistiocytic villitis	1.81%	0%
Thickened vessels	25.45%	23.63%

*P < 0.001, **P = 0.051.
